# Health-risk behaviors among Iranian university students, 2019: a web-based survey

**DOI:** 10.1186/s13690-020-00514-y

**Published:** 2020-12-09

**Authors:** Farhad Shekari, Peyman Habibi, Haidar Nadrian, Asghar Mohammadpoorasl

**Affiliations:** 1 Department of Statistics and Epidemiology, Faculty of Health, Tabriz University of Medical Sciences, Tabriz, Iran; 2 Department of Health Education and Promotion, Faculty of Health & Social Determinants of Health Research Center, Tabriz University of Medical Sciences, Tabriz, IR Iran; 3 Research Center of Psychiatry and Behavioral Sciences, Tabriz University of Medical Sciences, Tabriz, Iran

**Keywords:** Web-based survey, Latent class analysis, Risky behaviors, Students

## Abstract

**Background:**

High-risk behaviors are among the most serious threats for the physical and mental health of adolescents and young adults. Our aims in this study were to investigate the subgroups of students based on risky behaviors and to identify the prevalence rate of these subgroups.

**Methods:**

This cross-sectional web-based survey was conducted from July to August 2019 in Tabriz, Iran. We performed proportional sampling in all nine universities of the city, according to the number of students in each university. Applying an online survey questionnaire, the data were collected from 3649 students and analyzed using Latent Class Analysis.

**Results:**

For total sample, standardized prevalence rates of cigarette smoking, hookah use, alcohol consumption, substance abuse and unsafe sex were 18.5 (Confidence Interval (CI) 95%: 17.3–19.8), 9.1 (CI 95%: 8.2–10.1), 9.2 (CI 95%: 8.3–10.2), 8.3 (CI 95%: 7.4–9.3) and 14.5 (CI 95%: 13.3–15.7), respectively. Three latent classes of risky behaviors were determined among students: a) low risk b) smoking and c) high risk. About 18% of boys and 1.5% of girls were in the high risk class. Cigarette smoking (18.5%, CI 95%: 17.3–19.8) and substance abuse (8.3%, CI 95%: 7.4–9.3) were the most and the least common risky behaviors among the students.

**Conclusion:**

In this we-based survey, a considerable number of students, particularly boys (18%), was at high-risk class, stressing the need for preventive interventions for this group of youth. Our findings are beneficial for planning and development of risky-behavior preventive strategies to prevent high-risk behaviors among college students.

## Background

Performing risky behaviors, individuals expose themselves potentially to risks of harm [1]. High-risk behaviors are associated with increased risks of chronic diseases, premature mortality and disability, and have a negative impact on the physical and mental health of individuals [2]. Risky behaviors are also one of the most serious risk factors for adolescent and young people’s (12–18 years old) physical and mental health. Tobacco use, alcohol consumption, high-risk sexual behaviors, and drug abuse are among the most risky behaviors which may increase the likelihood of harmful physical, psychological and social consequences for individuals [3]. Drug use, alcohol consumption and risky sexual behaviors account for 2, 7, and 4% of disability-adjusted life years (DALY), respectively, among individuals with 15 to 24 years of age [4]. Considering their negative consequences, high-risk behaviors are among the most important areas of research in youth studies.

The prevalence rate of some risky behaviors is reported to be high among university students who constitute a large part of young population [5, 6]. Among Asian countries, high-risk behaviors are prevalent among adolescent and young populations in Thailand, Saudi Arabia, and the Middle East [7–9]. The high prevalence rate of such behaviors among Iranian students has also been reported. For example, a study conducted in 2017 among Iranian university students showed that among all 13.5% smoked cigarette, 7.8% drank alcohol, 4.9% had drug abuse and 7.8% had unprotected sex [10].

Analyzing youth subgroups in terms of risky behaviors may provide health care providers and policy makers with the opportunity to identify those who share the same characteristics based on high-risk behaviors [11]. Such findings may be also beneficial while designing traffic injury prevention strategies for youth. For instance, individuals who are solely involved in drug abuse may have characteristics that are different from those involved in other high-risk behaviors. In previous Iranian studies that clustering method was used to investigate high-risk behaviors among students, different subgroups of students for high-risk behaviors are reported. In a study conducted in Tabriz, Iran, three subgroups of low-risk, cigarette and hookah smoker, and high-risk were identified for risky behaviors. According to the study, 3.7% of boys and 0.4% of girls were in the high risk group [12]. In another study conducted in Iran four subgroups of high-risk behaviors among students were identified including low-risk, smoking cigarette and hookah, risky sexual behavior (in girls) and risky sexual behavior and alcohol consumption (in boys), and high-risk. In this study, 13.3% of boys and 4.3% of girls had high-risk behaviors [13]. In Bushehr, another Iranian city, five subgroups of risky behaviors among students are also reported: low-risk, high-risk, somewhat low-risk, hookah consumption, and very high-risk. It is noteworthy that 7.7 and 2.5% of students had high-risk and very high-risk behaviors, respectively [14].

Compared to the traditional paper-based studies, in the studies where online or web-based questionnaires are used to collect data, respondents are more honest in answering to sensitive questions, like having sex or using drugs, due to anonymity [15–20]. All previous Iranian studies that examined high-risk behaviors among students have used a written questionnaire to collect data. As mentioned, in this method of data collection, there is the possibility for the respondents to answer the sensitive questions incorrectly. Considering this issue and keeping in mind the high prevalence rate of high-risk behaviors among Iranian students [10, 21–23], we conducted this web-based study to identify the subgroups of students based on risky behaviors, and to determine the prevalence rate of the subgroups among a representative sample of university students in Tabriz, Iran.

## Methodology

This web-based cross-sectional study was conducted in Tabriz from July to August, 2019. There are 9 universities in Tabriz. We performed proportional sampling in all nine universities of the city, according to the number of students in each university. In total, 3788 students completed the online questionnaire, of which 139 cases were incomplete. Finally, the data collected from 3649 students were analyzed. All participants were fulltime and nationally Iranian students.

A questionnaire was developed to evaluate high-risk behaviors among the students. All items were designed according to the scientific literature and using the opinions of experts, which had previously been used in other studies. To assess validity, the questionnaire was presented to 11 experts in the field of substance abuse and methodology and instrumentation, and five knowledgeable students, along with a response form for the quantitative comments on the relevancy and transparency of the questionnaire. To assess reliability, a pilot study was conducted on 30 students. After receiving the responses and revising the questionnaire, we designed the final questionnaire in the Google form.

All students were invited to participate in the study, and a short link to the questionnaire was provided to them to complete the questionnaire online. Telegram and Instagram applications were also used to get more students involved in the study. The administrators and representatives of the channels and groups of the students in Tabriz universities were identified and all were asked to place the questionnaire link in the associated channel or group so that the students can enter into the link and complete the questionnaire, in the case of being consent to participate in the study. Participation was voluntary, and the participants’ anonymity was ensured. To maintain the sampling portion in the universities, the number of study participants from each university was monitored as the questionnaires were completed. In the case of fulfilling the predetermined number of participants from a given university, the researchers then terminated sampling from that university and focused on the universities with insufficient sample size.

To assess risky behaviors among students, five questions with a dichotomous response format were developed. These questions were: 1) “Have you currently smoked cigarette?” 2) “Have you currently used hookah (At least once a week)?” 3) “Have you consumed alcohol in the last 30 days?” 4) “Have you ever experienced substance abuse?” and 5) Unsafe sex was assessed applying three separate questions; “In the case of having sexual relationship, have you consumed alcohol or drugs prior/while sexual intercourse?”, “Do you have sexual intercourse with multiple partners?” and “Do you regularly use condom while sexual intercourse?” Respondents who answer “yes” to at least one of the three questions were classified as having unsafe sex.

Latent class Analysis (LCA) with gender as a group variable was used to analyze data. To perform LCA, the five dichotomous variables were used to assess risk-taking behaviors among students, as a latent variable. To perform LCA, the models with 1 to 7 classes were considered and for each model Akaike information criterion (AIC) and Bayesian information criterion (BIC) were calculated. For all information criteria, a smaller value represented a more optimal balance of model fit and parsimony; thus, a model with the minimum AIC or BIC was selected. All analyses were performed using proc-LCA in SAS software version 9.2 (SAS Institute Inc., Cary, NC, USA). Because of the difference between the number of girls and boys and the frequency of risk-taking behaviors between two genders, we used direct standardization method to calculate the risk-taking behaviors for all the sample.

## Results

The age range of participants was 18–37 years (Mean ± standard deviation: 22.8 ± 3.7). More than half of the students were male (55.7%) and only 10.0% were married. The frequency of risk-taking behaviors is shown in Table 1. As our results showed, the frequency of smoking and having unsafe sex were higher compared to other risky behaviors. Also, risk-taking behaviors were more prevalent among male than female students.

**Table 1:** Frequency of Students Responding “Yes” to Questions about Risk-Taking Behaviors among Iranian University Students, 2019

Items	Male (*N* = 2034)		Female (*N* = 1615)		Total (Standardized prevalence for sex)
	n	% (CI 95%)	n	% (CI 95%)	% (CI 95%)
Smoking	734	36.4 (34.3–38.5)	121	7.6 (6.4–8.9)	18.5 (17.3–19.8)
Hookah use	359	17.7 (16.1–19.4)	62	3.9 (3.0–4.9)	9.1 (8.2–10.1)
Alcohol use	337	16.7 (15.1–18.4)	75	4.7 (3.7–5.8)	9.2 (8.3–10.2)
Substance abuse	299	15.3 (13.8–17.0)	65	4.1 (3.2–5.2)	8.3 (7.4–9.3)
Sexual risk behavior	408	20.5 (18.7–22.3)	168	10.7 (9.3–12.4)	14.5 (13.3–15.7)

Note. *CI* Confidence Interval

Based on the five dichotomous variables, there were 32 possible response patterns. The comparison of LCA models with different latent classes is presented in Table 2. We found that the three latent classes’ model was appropriate for both males and females, based on the model selection indices and the interpretability results of the models.

**Table 2:** Comparison of LCA Models with Different Latent Classes Based on Model Selection Statistics among Iranian University Students, 2019

Number of latent Classes	Number of parameters estimated	G^2^	df	AIC	BIC	Maximum log-likelihood
1	5	1806.28	53	1826.28	1888.28	− 6824.14
2	11	124.29	41	168.29	304.71	− 5983.15
3	17	48.75	29	116.75	327.58	− 5945.38
4	23	25.70	17	117.70	402.94	− 5933.86
5	29	13.34	5	129.34	488.98	− 5927.67
6	35	6.81	–	146.81	580.87	− 5924.41
7	41	6.86	–	170.86	679.32	−5924.43

Note. *LCA* latent class analysis, *AIC* Akaike Information Criterion, *BIC* Bayesian Information Criterion

The results of three classes LCA models for both male and female students are presented in Tables 3 and 4, respectively. As there is shown in the tables, nearly 59 and 18% of male students were in low risk and high risk for having risk-taking behaviors, respectively. Among female students, about 88% were at low risk and 1.5% were at high risk for performing risk-taking behaviors. Also, about 23% of male students and 10.6% of female students were in the cigarette smoker class.

**Table 3:** The three latent classes model of risk-taking behaviors among male Iranian University Students, 2019

	Latent class		
	Low risk	Cigarette smoker	High risk
Latent class prevalence	58.9	23.2	17.9
Item-response probabilities	Probability of a “Yes” response		
Smoking	0.000	**0.934**^**a**^	**0.818**
Hookah use	0.054	0.236	**0.507**
Alcohol use	0.022	0.140	**0.676**
Substance abuse	0.021	0.204	**0.543**
Sexual risk behavior	0.110	0.107	**0.638**

Note. The probability of a “No” response can be calculated by subtracting the item-response probabilities shown above from 1

^a^Item-response probabilities > 0.5 in bold to facilitate interpretation

**Table 4:** The three latent classes model of risk-taking behaviors among female Iranian University Students, 2019

	Latent class		
	Low risk	Cigarette smoker	High risk
Latent class prevalence	87.9	10.6	1.5
Item-response probabilities	Probability of a “Yes” response		
Smoking	0.008	**0.523**^**a**^	**0.895**
Hookah use	0.006	0.240	**0.556**
Alcohol use	0.010	0.214	**0.999**
Substance abuse	0.000	0.296	**0.743**
Sexual risk behavior	0.073	0.264	**1.000**

Note. The probability of a “No” response can be calculated by subtracting the item-response probabilities shown above from 1

^a^Item-response probabilities > 0.5 in bold to facilitate interpretation

## Discussion

Our aim in this web-based study was to identify subgroups of students based on risky behaviors, and to determine the prevalence of these subgroups among a representative sample of students in Tabriz, Iran. Our results showed that the frequency of risky behaviors, namely smoking, hookah use, alcohol use, substance abuse, and having unsafe sex were 18.5, 9.1, 9.2, 8.3 and 14.5%, respectively. The results of a previously published meta-analysis showed an approximate prevalence rate of 42% for high-risk sexual behaviors among Ethiopian students [24]. In a study among American students, the prevalence rates of alcohol consumption, cigarette and hookah smoking were 44, 31 and 22%, respectively [25]. Another study among Canadian students revealed that 55% smoked cigarette, 62% consumed alcohol, 36% had drug abuse, and 28% had high-risk sexual intercourse [26]. The reason for the low prevalence of high-risk behaviors among Iranian students compared to other countries may be due to cultural-religious beliefs in Iran, as well as the religious prohibition of alcohol consumption and the legal prohibition of alcohol and drug abuse.

The results of a study among students in Khorramabad, Iran, showed that the prevalence rates of smoking cigarette, drug abuse, and alcohol consumption were 3.7, 2.4, and 5.5%, respectively [27]. Another study conducted on Tehran University students showed that the prevalence rates of alcohol, drug abuse, and high-risk sexual behaviors were 4.6, 2.3, and 5.6%, respectively [28]. Another study on the prevalence of high-risk behaviors among students in Rudan, Iran, demonstrated that the prevalence rates of drug use, alcohol consumption, and high-risk sexual behaviors were 5.5, 4.9, and 6.6%, respectively [29]. Another study conducted in 2011 in Tabriz, Iran, showed that the prevalence rates of smoking cigarette, smoking hookah, alcohol consumption, drug abuse, and high-risk sexual intercourse were 15.8, 8.5, 8, 7.6, and 10.8%, respectively [13]. As evident, all these studies reported lower levels of risk-taking behaviors compared to our findings in the present study, which may be attributed to the use of online questionnaire in the present study. As evidences suggest, the quality of responses in sensitive issues to online questionnaires is better than that of paper questionnaires [15–20]. As risky behaviors may increase over time, we should also consider the differences in the time of conducting the present study and those of previous studies in Iran.

Overall, the results of our study showed that smoking cigarette (total: 18.5%, boys: 36.4%, girls: 7.6%) and drug abuse (total: 8.3%, boys: 15.3%, girls: 4.1%) were the most and the least common risky behaviors among Iranian college students, respectively. Our results also showed that boys had more risky behaviors than girls. These findings were in line with those reported in other studies conducted on Iranian students [29–31]. The low prevalence of risky behaviors among Iranian female students compared to male students can be due to different cultural and social expectations and greater freedom of boys which may facilitate the inclination of boys towards risky behaviors [32, 33]. These results indicate the greater need for preventive interventions of risky behaviors in male students. Public health policymakers should also plan to develop policies on smoking cessation interventions especially for men within communities.

A useful preventive measure is taking into account the concurrency of high-risk behaviors. As previous research suggests, engagement in one high-risk behavior is associated with engagement in other high-risk behaviors [34]. Numerous studies have also shown the concurrency of cigarette smoking and hookah smoking [35], smoking and alcohol consumption [36], cigarette smoking and drug abuse [37], and high-risk sexual behaviors and alcohol and drug abuse [38]. In the present study, our findings showed that the students of both genders had three subgroups in terms of risk taking behaviors including low-risk, smoking cigarette and high-risk, with the prevalence rates of 58.9, 23.2 and 17.9% in boys and 87.9, 10.6 and 1.5% in girls, respectively. The results of all above-mentioned studies shed light to the co-occurrence and the dynamic change of high-risk behaviors as a core category while designing interventions to prevent and reduce risky behaviors among adolescents and youth within communities.

In previous studies that used the clustering method to investigate high-risk behaviors among students, different subgroups of students with high-risk behaviors were identified. A study in Tabriz showed three subgroups or classes of risky behaviors among students, including low-risk, smoking cigarette and hookah, and high-risk [12]. According to this study, 3.7% of boys and 0.4% of girls were in the high-risk class, which are lower than those found in the present study. In the study conducted by Safiri et al. (12), high-risk behaviors were less prevalent among girls than boys, which is in line with the findings of our study. In another study conducted in Tabriz in 2011, four subgroups of high-risk behaviors among students were identified including low-risk, smoking cigarette and hookah, risky sexual behavior in girls and risky sexual behavior and alcohol consumption in boys, and high-risk. According to this study, 13.3% of boys and 4.3% of girls had high-risk behaviors [13]. As mentioned above, a reason for such inconsistencies could be attributed to the quality of responses due to online questionnaires in the present study.

Studies applying the LCA method to investigate the concurrency of high-risk behaviors among students in different societies have shown different patterns of behavior among university students. For instance, a study on the US students who involved in tobacco use, drug abuse, and alcohol consumption showed five latent classes for the behaviors [25]. According to this study, being a boy increased the chance of placement in high-risk class. Additionally, 61.8% of the students were in non/low user class and 5.6% were in poly-substance user class. Another similar study in Canada identified three latent classes of the behavior: Normal (65.7%), relatively healthy (14.5%), and high-risk (19.8%) [26]. Similarly, Chiauzzi et al. in the US found four classes for the behaviors, namely low-risk alcohol consumption/low prevalence of drug abuse (46.0%), lower alcohol consumption/moderate prevalence of drug abuse (20.2%), moderate-risk alcohol consumption/moderate prevalence of drug abuse (13.6%), and high-risk alcohol consumption/high prevalence of drug abuse (20.2%) [39]. The higher prevalence rates of high-risk classes in these studies compared to our study may be due to geographical and cultural differences and the type of norms.

There were several limitations in the present study. Although we believe that the students provided us with somewhat honest answers, compared to paper-based surveys, our results were still based on self-report of the participants. According to previous research, in the studies that online questionnaires are used to collect data, usually there is a problem with low response rate [40, 41], and this may come true for our study, yet we do not know how much was the number of students who viewed the questionnaire link but did not answer to the questions. Thus, we cannot determine the response rate. Another limitation of our web-based study is participation bias, which may distort the results [42], because not all students with internet access may have a social network account and wish to participate in the study.

## Conclusion

In this we-based survey, three subgroups of risky behaviors were identified among the students of both genders. There was also a considerable percentage of students, particularly boys, at high-risk class, stressing the need for preventive interventions for this group of youth. Our findings are beneficial for planning and development of risky-behavior preventive measures to prevent high-risk behaviors among college students.

## Data Availability

Please contact the corresponding author for data requests.

## Abbreviations

LCA: Latent class Analysis
AIC: Akaike information criterion
BIC: Bayesian information criterion
